# Danshenol A inhibits TNF-α-induced expression of intercellular adhesion molecule-1 (ICAM-1) mediated by NOX4 in endothelial cells

**DOI:** 10.1038/s41598-017-13072-1

**Published:** 2017-10-11

**Authors:** Wenwen Zhao, Haitao Feng, Shuhui Guo, Yantao Han, Xiuping Chen

**Affiliations:** 1 Qingdao University Medical College, 308 Ningxia Road, Qingdao, Shandong 266021 China; 2 State Key Laboratory of Quality Research in Chinese Medicine, Institute of Chinese Medical Sciences, University of Macau, Macao, China

## Abstract

ICAM-1 overexpression and subsequent adhesion of leukocytes to endothelial cells play critical roles in the early stage of atherosclerosis. Danshenol A (DA) is an abietane-type diterpenoid isolated from traditional Chinese herb *Salvia miltiorrhiza* Bunge. The mechanisms under its regulation of adhesion of molecular expression are explored. Here, the effect of DA on TNF-α-induced ICAM-1 expression was investigated in endothelial cells. TNF-α-induced ICAM-1 expression and subsequent adhesion of monocytes, as well as elevated reactive oxygen species (ROS) generation and NOX4 expression were all significantly reversed by DA, siNOX4 and NOX4 inhibitor GKT137831. Furthermore, TNF-α-induced ICAM-1 expression, which was increased via IKKβ/IκBα-mediated activation of NF-κB p65, was also inhibited by DA. Interestingly, NOX4 overexpression suppressed the ICAM-1 expression, and this finding may be ascribed to the activation of Nrf-2. Additionally, NF-κB inhibitor PDTC, siNOX4, or DA can decrease the TNF-α-induced ICAM-1 expression and suppress the adhesion of monocytes. In all, DA inhibited TNF-α-induced ICAM-1 expression and subsequent monocyte adhesion to endothelial cells through the NOX4-dependent IKKβ/NF-κB pathway. Besides, NOX4 played dual role in regulating ICAM-1 expression *via* diverse signal pathway. This novel bioactivity will make DA a good candidate to be further explored for therapeutic or preventive application for atherosclerosis.

## Introduction

Atherosclerosis is known as a chronic inflammatory disorder^1^. As an important proinflammatory cytokine, TNF-α activates endothelial cells at the site of inflammation, leading to the upregulation of endothelial cell adhesion molecules, such as vascular cell adhesion molecule-1 (VCAM-1)^2^, intercellular adhesion molecule (ICAM-1)^3^, and endothelial leukocyte adhesion molecule-1 (E-selectin)^4^. The increased adhesion molecules mediate local leukocyte accumulation, adherence, and subsequent transmigration into subendothelial space, which is an early step in atherosclerosis^5^.

Increasing evidence has shown that TNF-α leads to oxidative stress by increasing reactive oxygen species (ROS) production^6,7^. The expression of adhesion molecules is also widely considered to be activated in an oxidative stress scenario^8^. As such, we can reasonably speculate that ROS is responsible for the expression of TNF-α-induced adhesion molecules and the subsequent leukocyte accumulation. The multiple sources of ROS *in vitro* include NADPH oxidases, mitochondria, xanthine oxidase (XO), and lipoxygenase (LOX)^9–13^. In the past decades, the mitochondrion has always been considered as the main source of ROS in vascular cells^12,14^. Recently, the NADPH oxidase (NOXs) family, as an important source of ROS outside the mitochondria, has been implicated in the pathophysiology of vascular diseases^15,16^. Among the seven NOXs members (NOX1-5, DUOX1/2), NOX1, NOX2, and NOX4 are the three main NADPH oxidases highly expressed in endothelial cells. At present, NOX2 and NOX4 are reported to be primarily responsible for ROS generation and have received much attention as regulators of vascular diseases, such as atherosclerosis and hypertension^9,17^. By contrast, recent findings suggested that NOX4 might also be vascular protective. NOX4 overexpression promotes proliferation and migration of endothelial cells but reduces serum-deprivation-induced apoptosis in human microvascular endothelial cells^18^. Furthermore, transgenic mice with endothelium-specific NOX4 overexpression exhibited enhanced vasodilation and reduced blood pressure^19^. More recently, endothelial NOX4 demonstrated protection of ApoE^−/−^ mice from atherosclerotic lesions^20^.

Several lines of evidence have shown that the expression of TNF-α-induced molecules is regulated at the gene level by activation of transcription factors. These transcription factors, including Nrf-1, Nrf-2, and NF-κB p65, are redox sensitive and identified to be involved in the regulation of the gene expression of leukocyte-adhesion molecules and cytokines^21–23^.

*Salvia miltiorrhiza* Bunge (Danshen) is a traditional Chinese herb with a lot of properties that could improve our health. In pharmaceutical field, it has been a continuous source of therapeutic agents for cerebrovascular disorders while in food field, danshen is used as food additives to make herbal tea, soups, seasoner and beverages. Danshen is currently going through trials and may even become the first traditional Chinese remedy to gain approval by the U.S. Food and Drug Administration (FDA)^24^.

**Figure 1:**
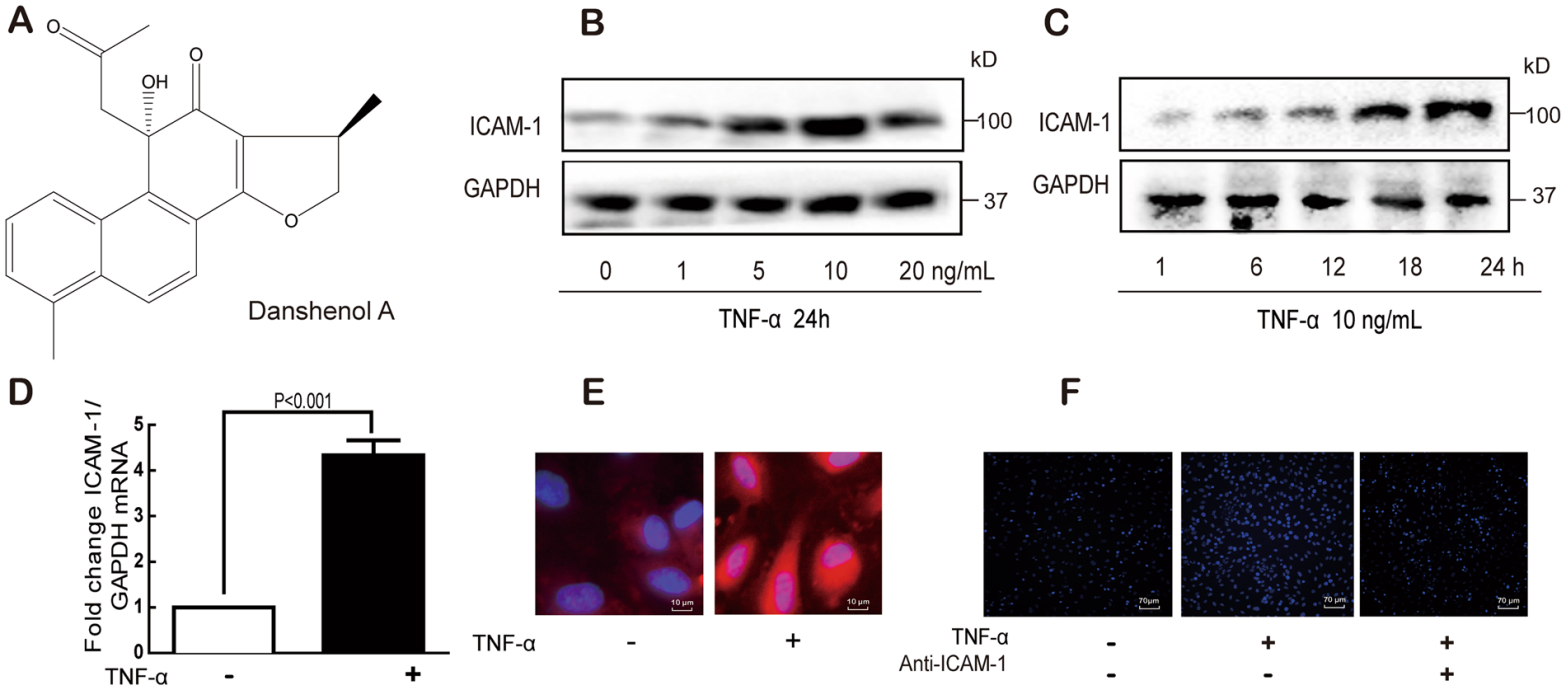
TNF-α induces ICAM-1 expression and monocyte–HUVEC adhesion. Chemical structure of DA (**A**). Cells were treated with TNF-α, and the ICAM-1 protein expression was detected by Western blot (**B** and **C**). Cells were treated with TNF-α (10 ng/mL) for 24 h; ICAM-1 mRNA and protein expression were detected by real-time PCR (**D**) and immunofluorescence staining (**E**), respectively. Cells were treated with TNF-α (10 ng/mL) for 24 h, and the adhesion of monocytes to endothelial cells was determined with or without ICAM-1 antibody (**F**). Data were presented as mean ± SD of three independent experiments.

Danshenol A (Fig. 1A) is an abietane-type diterpenoid isolated from Danshen in 1997^25^. Compared with well-known tanshinones such as tanshinone IIA, tanshinone I, and cryptotanshinone, its biological effects have been limited reported. Recently, studies focused on DA are paid more and more attentions. One paper reported that DA has a strong inhibitory effect on aldose reductase (AR)^25^. Also some study showed that DA has anti-inflammatory properties superior to those of tanshinone IIA^26^ hinting that DA has potential for anti-atherosclerosis. One early phase of atherosclerosis involves the recruitment of inflammatory cells from the circulation and their transendothelial migration. This process is predominantly mediated by cellular adhesion molecules^27^.

ICAM-1, one of the largest families of cell adhesion molecules plays an important role in the process of atherosclerosis^28^. In the present study, we investigated the effects and potential mechanisms of DA on the TNF-α-induced ICAM-1 expression in human umbilical vein endothelial cells (HUVECs). We found that DA inhibited the TNF-α-induced ICAM-1 expression by suppressing the ROS-generating NOX4. Furthermore, NOX4 played a dual role in regulating the ICAM-1 expression induced by TNF-α.

## Results

### DA inhibited TNF-α-induced ICAM-1 expression and subsequent monocyte adhesion

**Figure 2:**
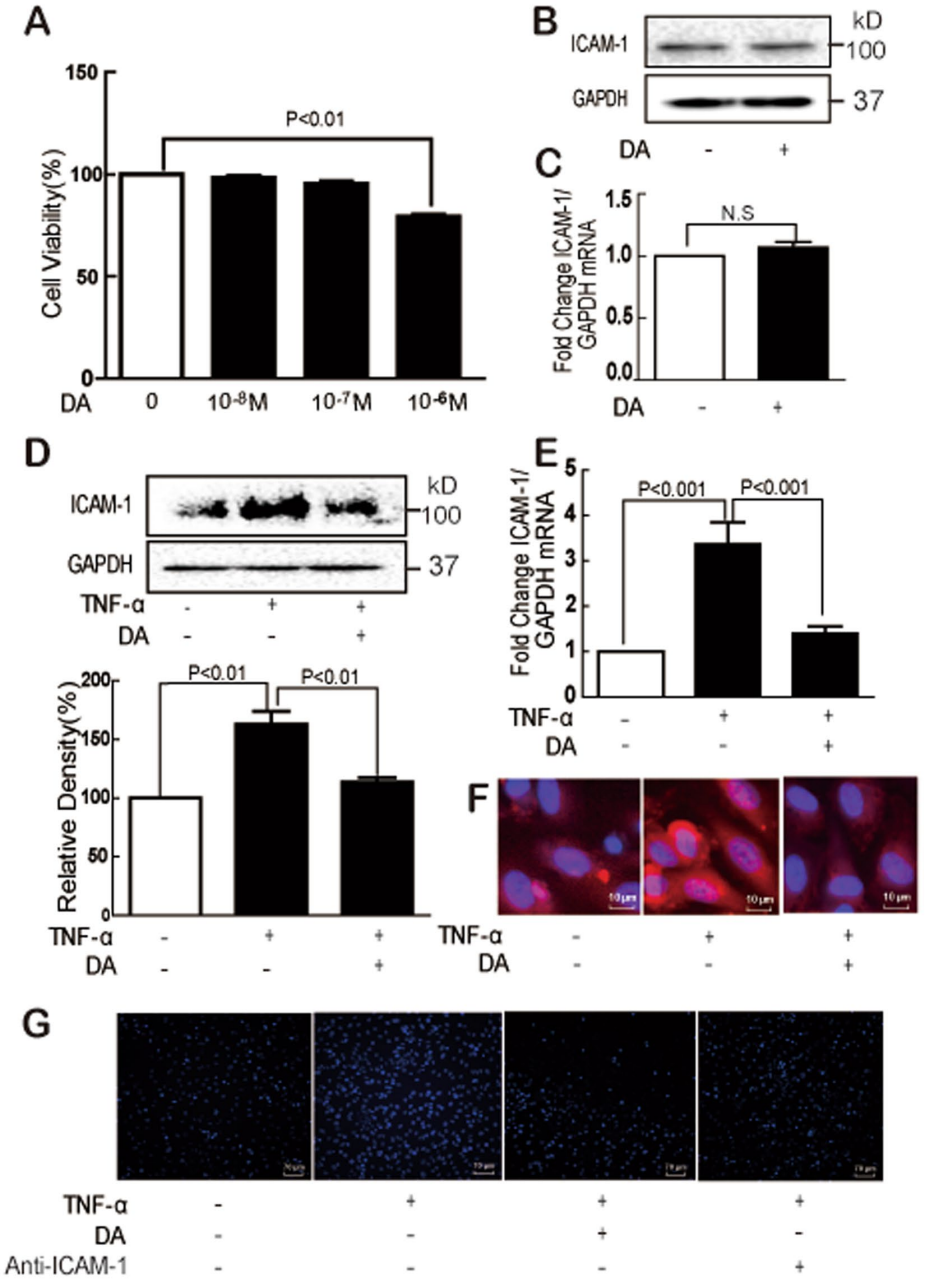
DA inhibited TNF-α-induced ICAM-1 expression and monocyte–HUVEC adhesion. Cells were treated with DA, and cytotoxicity was determined by MTT assay (**A**). Cells were treated with DA (10 nM) for 24 h; ICAM-1 expression was detected by Western blot (**B**) and RT-PCR (**C**). Cells were treated with TNF-α (10 ng/mL) for 24 h in the absence or presence of DA (10 nM) pretreatment for 1 h; ICAM-1 protein and mRNA expression was detected by Western blot (**D**), immunofluorescence staining (**F**), and RT-PCR (**E**). The effect of DA on the adhesion of monocytes to endothelial cells was determined (**G**). Data were presented as mean ± SD of three independent experiments. NS indicates no significance.

We first characterized the effects of TNF-α on ICAM-1 expression in endothelial cells. TNF-α significantly increased the ICAM-1 protein expression in a concentration- and time-dependent manner (Fig. 1B and C). The mRNA expression of ICAM-1 was also upregulated by TNF-α (Fig. 1D). Immunofluorescene assay showed that significant red fluorescence was observed in the TNF-α-treated endothelial membrane (Fig. 1E). Moreover, TNF-α treatment considerably increased the adhesion of monocytes to endothelial cells, but this adhesion was significantly inhibited by the anti-ICAM-1 antibody (Fig. 1F). The cytotoxicity of DA to endothelial cells was then evaluated, and low concentration of DA (100 nM) showed no cytotoxicity (Fig. 2A). To minimize the cytotoxicity and test the efficacy of DA, 10 nM DA was selected in the following study. DA (10 nM) alone showed no effect on the ICAM-1 expression at both mRNA and protein levels (Fig. 2B and C). Furthermore, TNF-α induced all the alterations mentioned above (Fig. 1), which were significantly reversed by DA or anti-ICAM-1 antibody pretreatment (Fig. 2D–G).

### DA restored TNF-α-induced redox imbalance by inhibiting TNF-α-induced NADPH oxidase activity

**Figure 3:**
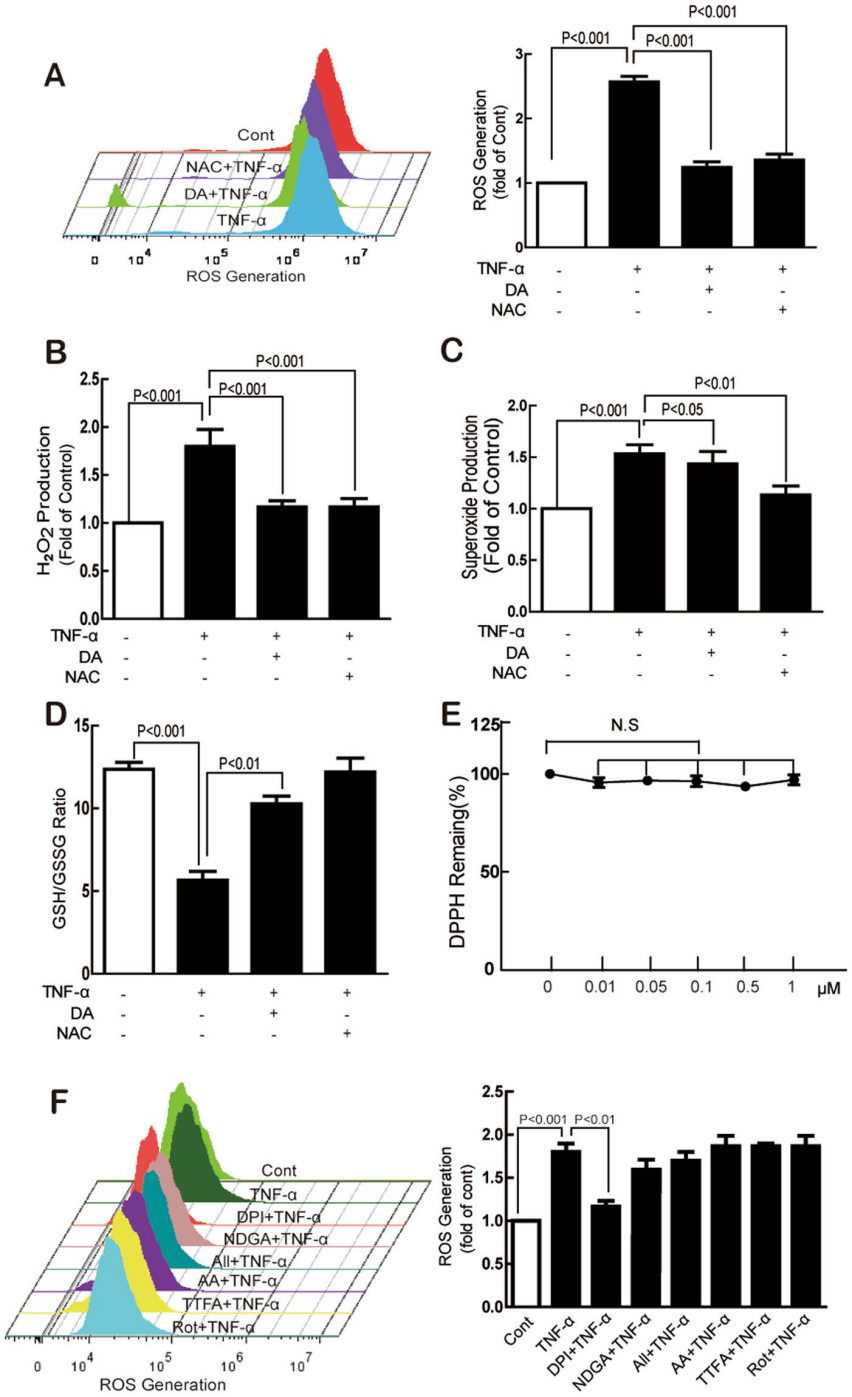
DA restored TNF-α-induced endothelial redox imbalance by inhibiting TNF-α-induced NADPH oxidase activity. Cells were treated with TNF-α (10 ng/mL) for 1 h with or without DA (10 nM), NAC (5 mM), Rot (20 μM), TTFA (10 μM), AA (5 μM), DPI (1 μM), All (10 μM), or NDGA (10 μM) pretreatment for 1 h, and the generation of ROS, H_2_O_2_, and O_2_^•−^ was determined by DCFH_2_-DA (**A** and **F**), Amplex Red (**B**), and DHE (**C**), respectively. GSH/GSSG levels were measured using commercial kits (**D**). DPPH radical scavenging activity was determined in a cell-free system (**E**). Data were presented as mean ± SD of three independent experiments.

Compared with the control, TNF-α induced approximately three fold ROS generation, which was almost completely inhibited by DA or NAC (Fig. 3A). Similar to NAC, DA significantly suppressed TNF-α-induced H_2_O_2_ production whereas DA had almost no effect on TNF-α-induced O2^•−^ production (Fig. 3B and C). Additionally, TNF-α-induced decrease of the GSH/GSSG ratio was significantly reversed by DA and NAC (Fig. 3D). However, DA showed no direct free radical scavenging capacity with DPPH (Fig. 3E).

To explore the specific sources of ROS generation induced by TNF-α, several inhibitors were applied. As shown in Fig. 3F, TNF-α-induced ROS production was significantly inhibited by DPI, a NADPH oxidase inhibitor. By contrast, ALL, Rot, TTFA, NDGA, and AA showed no obvious effects.

### DA inhibited TNF-α-induced NOX4 expression

**Figure 4:**
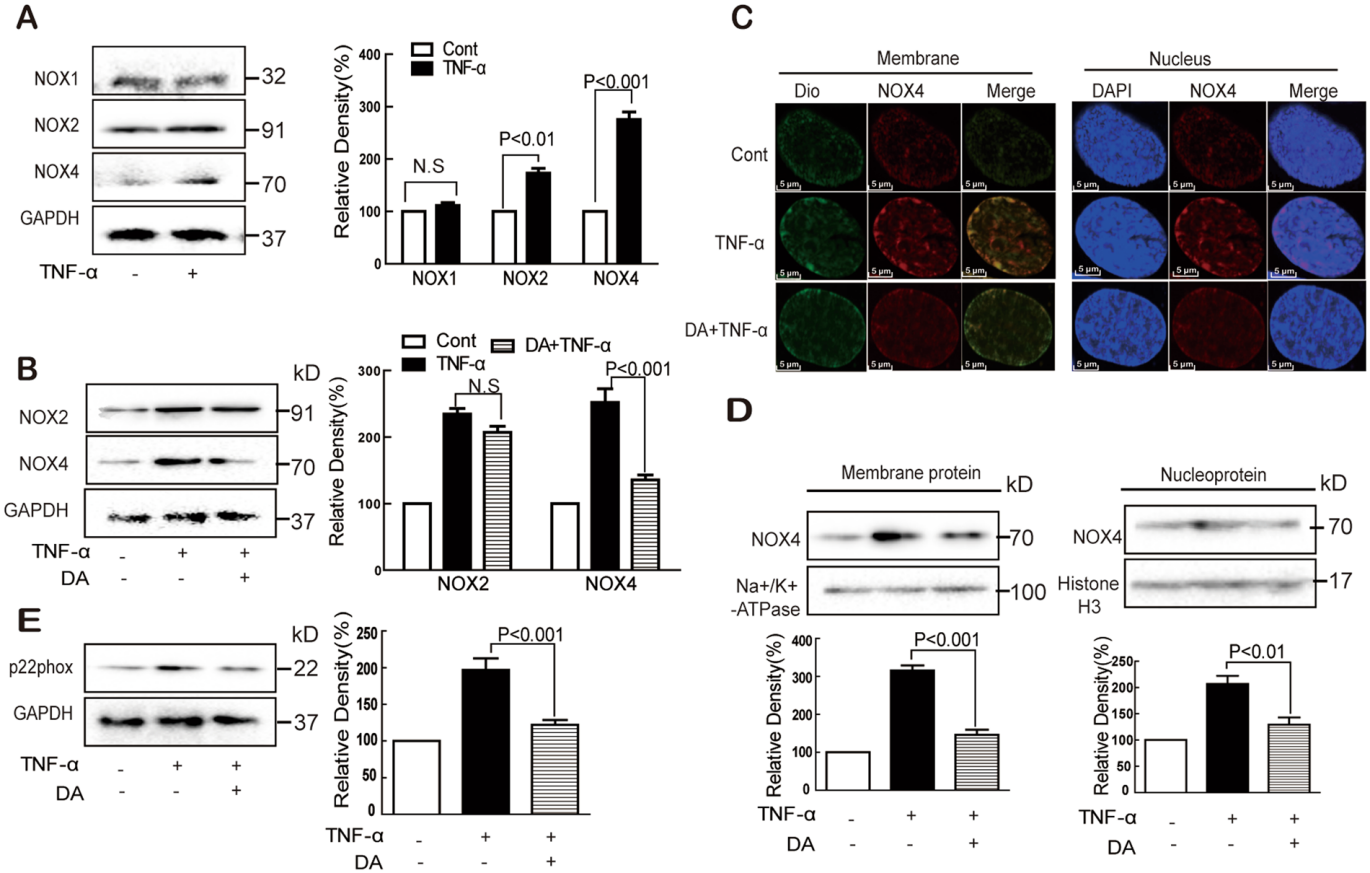
DA inhibited TNF-α-induced NADPH oxidase expression. Cells were treated with TNF-α (10 ng/mL) for 24 h with or without DA (10 nM) pretreatment for 1 h, and the protein expression of NOX1, NOX2, NOX4, and p22phox was detected by Western blot (**A**,**B**,**D** and **E**) or immunofluorescence (**C**). Data were presented as mean ± SD of three independent experiments. NS indicates no significance.

NOX1, NOX2, and NOX4 are highly expressed in endothelial cells^29,30^. TNF-α upregulated the protein expression of NOX4 and NOX2 but not NOX1 (Fig. 4A). DA treatment significantly inhibited the TNF-α-induced NOX4 without affecting NOX2 (Fig. 4B). In addition, TNF-α-induced NOX4 protein expression both on the membrane and in the nucleus was partly inhibited by DA (Fig. 4C and D). The TNF-α-induced expression of p22phox, an essential adaptor protein for functional NOX4, was also decreased by DA pretreatment (Fig. 4E).

### NOX4 knockdown inhibited TNF-α-induced ICAM-1 expression

**Figure 5:**
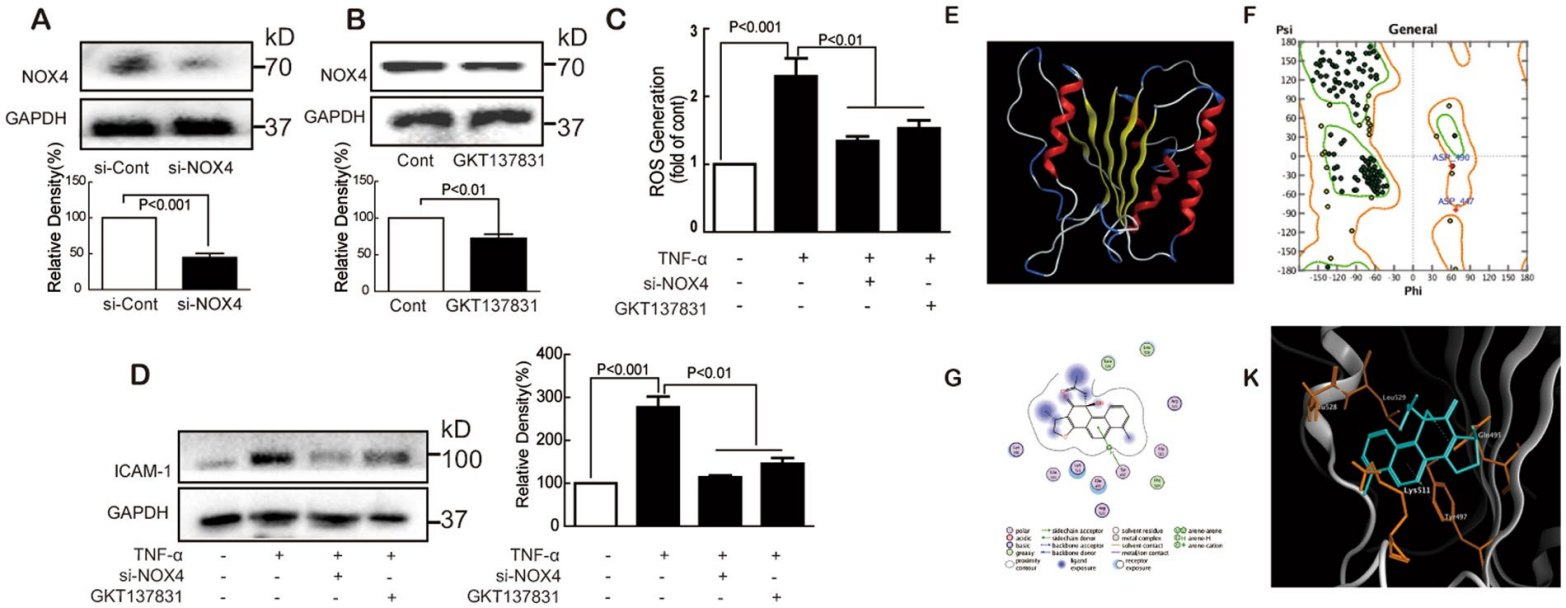
Knockdown of NOX4 inhibited TNF-α-induced ICAM-1 expression in endothelial cells. NOX4 was silenced by siNOX4 or inhibitor GKT137831 (**A** and **B**); After NOX4 was silenced, cells were treated with TNF-α (10 ng/mL) and ROS generation was detected by flow cytometry (**C**) and ICAM-1 was determined by Western blot (**D**); The constructed model of NOX4 (**E**); Ramachandran plot for NOX4 (**F**); Dark green dots represent the residues in most favored regions, yellow dots represent the residues in allowed regions; The ligand interaction of DA with NOX4 (**G**); The binding model of DA with NOX4 (**K**). The ligand is colored in blue, and the surrounding residues in the binding pockets are colored in orange. Data were presented as mean ± SD of three independent experiments.

To further confirm the role of NOX4 in the TNF-α-induced ICAM-1 expression, NOX4 expression was decreased by siNOX4 or inhibitor GKT137831 (Fig. 5A and B). Then we found that ROS production (Fig. 5C) in response to TNF-α was significantly reduced in both siNOX4 and GKT137831 groups. Furthermore, TNF-α-induced expression of ICAM-1 levels was also decreased accompanied with decreased NOX4 (Fig. 5D). To investigate the direct inhibition of DA with NOX4, docking simulation studies were carried out. The crystal structure of NOX4 is not available, the 3D structures of NOX4 was constructed by homology modeling and the NOX4 model was displayed in Fig. 5E and F based on the X-ray crystal structure of NADPH binding domain of gp91(PDB3A1F) and the amino acid sequence of NOX4 (NP_056575.1). The ramachandran plot of NOX4 model suggested the good quality of this model. Then, we docked DA to NOX4. The docking score is −5.77 kcal/mol. DA bound at the entrance of the active site and make contacts with the catalytic residues (Tyr497, Gln495, Lys511 and Leu529). DA also developed a H-^π^ interaction with residue Tyr497 and a hydrogen bond with the residue Gln495 (Fig. 5G and K). Docking simulation studies indicates that DA interact with NOX4 through hydrogen bonds interactions.

### DA inhibited TNF-α-induced NF-κB activation

**Figure 6:**
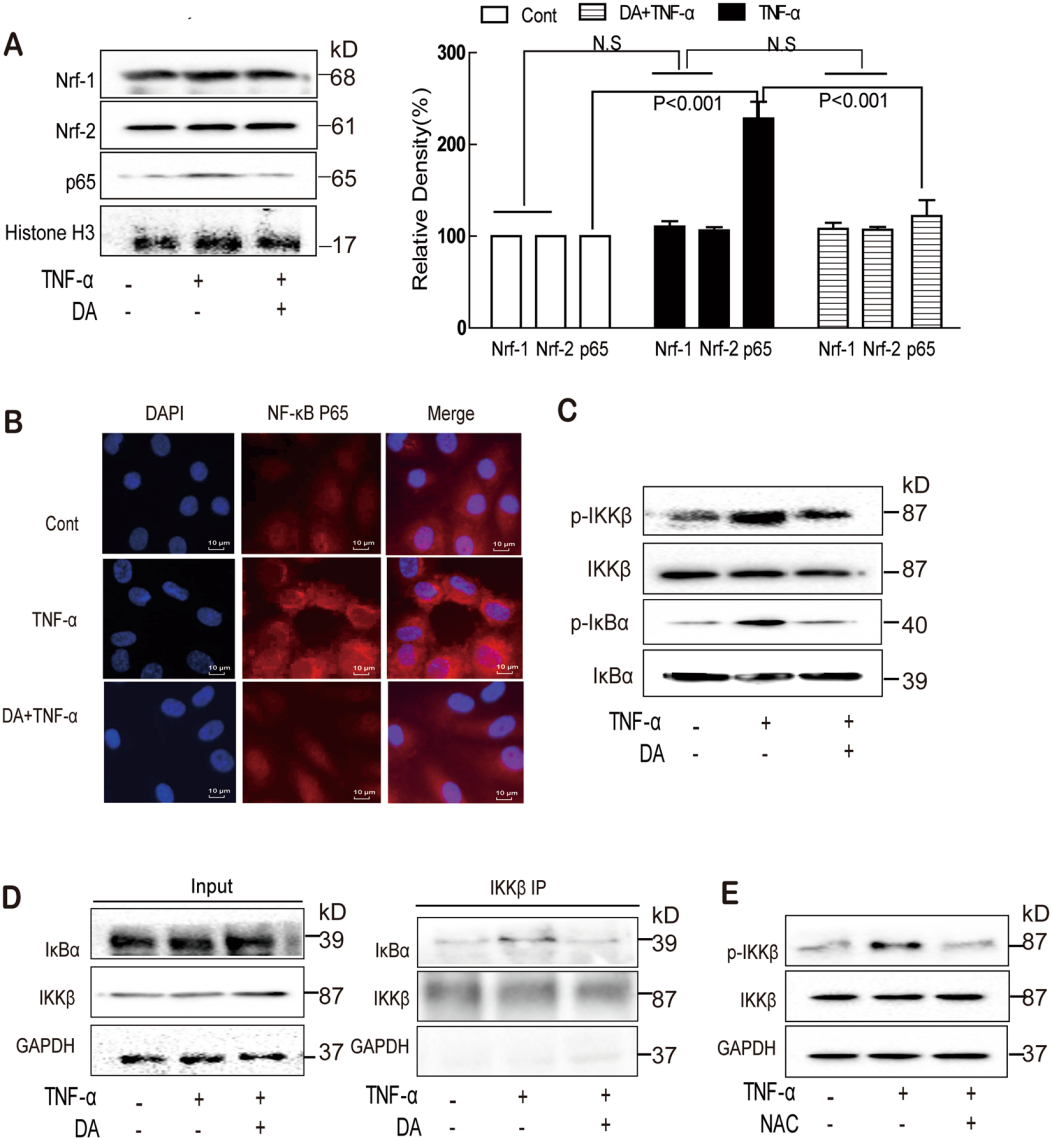
DA inhibited NF-κB pathways in endothelial cells. Cells were treated with TNF-α (10 ng/mL) for 1 h with or without DA (10 nM) pretreatment; the expression of Nrf-1, Nrf-2, and NF-κB p65 in the nucleus was detected by Western blot (**A**) and immunofluorescence staining (**B**). The expression of *p*-IκBα, IκBα, *p*-IKKβ, and IKKβ was also determined by Western blot (**C**). The interaction of IκBα and IKKβ was determined by co-immunoprecipitation assay (**D**). Cells were treated with TNF-α (10 ng/mL) for 1 h in the absence or presence of NAC (5 mM) for 1 h; the expression of IKKβ was detected by Western blot (**E**). Data were presented as mean ± SD of three independent experiments. NS indicates no significance.

NF-κB p65, Nrf-1, and Nrf-2 are oxidative-stress-sensitive transcription factors^31^. TNF-α treatment exhibited no effect on Nrf-1 and Nrf-2 expression but induced NF-κB p65 nuclear translocation, which was blocked by DA (Figs 6A and 8B). IKKβ is a kinase of the IKK family, and it can dissociate the IκBα/NF-κB complex to release active NF-κB into the nucleus^32,33^. As shown in Fig. 6C, increased phosphorylation of IKKβ and IκBα was observed in endothelial cells after TNF-α stimulation, which was significantly suppressed by DA. In addition, the TNF-α-induced interaction of IKKβ with IκBα was prevented by DA (Fig. 6D). Moreover, NAC can significantly inhibit the TNF-α-induced IKKβ phosphorylation hinting that IKKβ was downstream of ROS (Fig. 6E).

### NOX4 overexpression decreased TNF-α-induced ICAM-1 expression

**Figure 7:**
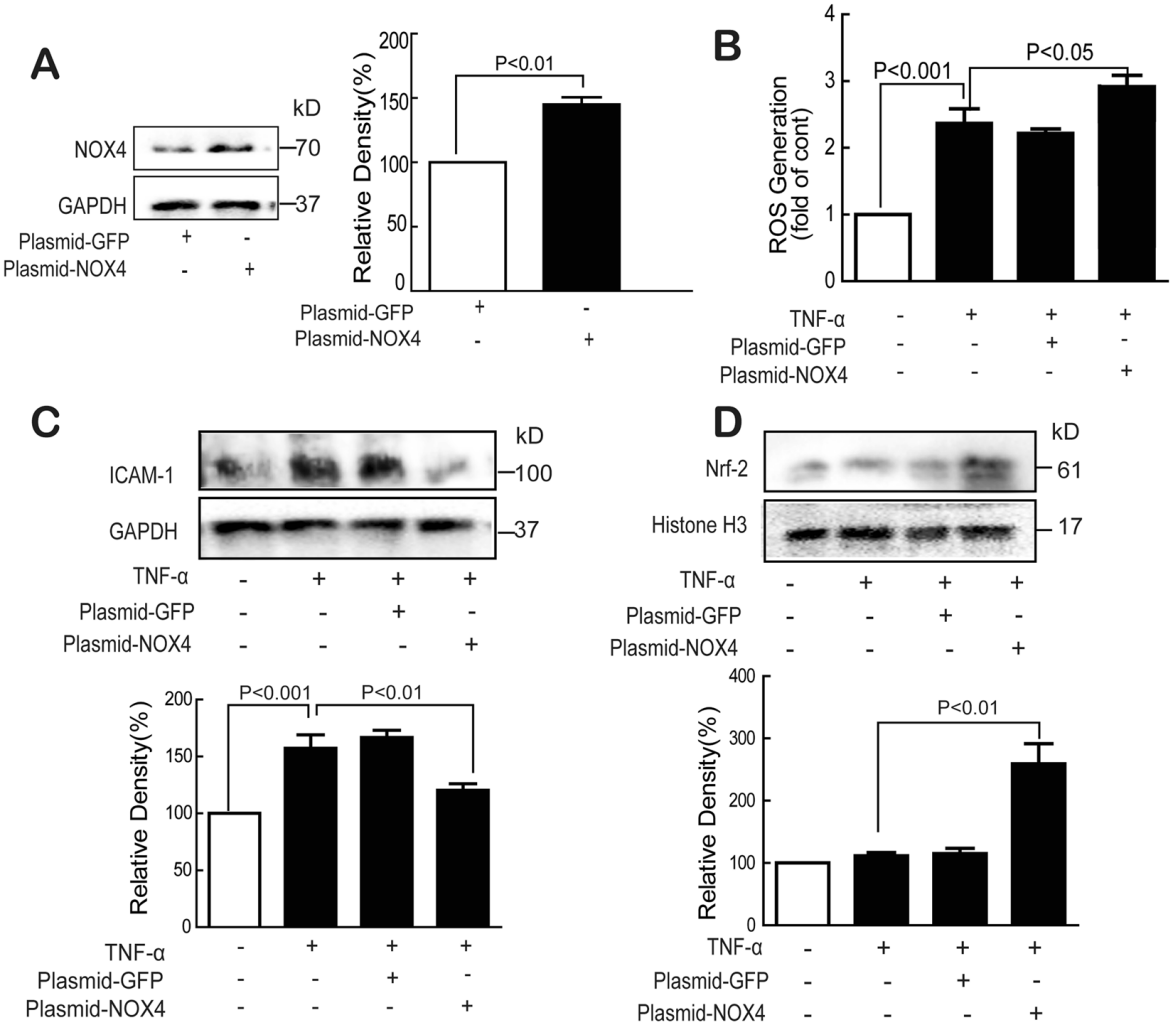
Overexpression of NOX4 inhibited TNF-α-induced ICAM-1 expression. Normal and NOX4 overexpressed cells (**A**) were treated with TNF-α (10 ng/mL) for 24 h, and the expression of NOX4, ICAM-1 and nuclear Nrf-2 (**A**,**C** and **D**), ROS generation (**B**) were determined by Western blot and flow cytometry. Data were presented as mean ± SD of three independent experiments.

To further explore the role of NOX4, full-length coding regions of NOX4 cDNA were cloned into a plasmid vector and transfected into HUVECs (Fig. 7A). As expected, TNF-α-induced ROS generation was further enhanced in NOX4-overexpressed cells (Fig. 7B). Interestingly, TNF-α treatment did not upregulate but downregulate the ICAM-1 expression in NOX4-overexpressed cells (Fig. 7C). Besides, although TNF-α exhibited no obvious effect on the nuclear Nrf-2 expression in normal cells (Fig. 6A), in NOX4-overexpression cells, nuclear Nrf-2 expression was obviously increased (Fig. 7D). We speculated that NOX4 overexpression induced Nrf-2 activation to inhibit TNF-α-induced ICAM-1 expression. We will do further research in the future.

### DA suppressed TNF-α-induced ICAM-1 expression via NOX4-dependent NF-κB pathway

**Figure 8:**
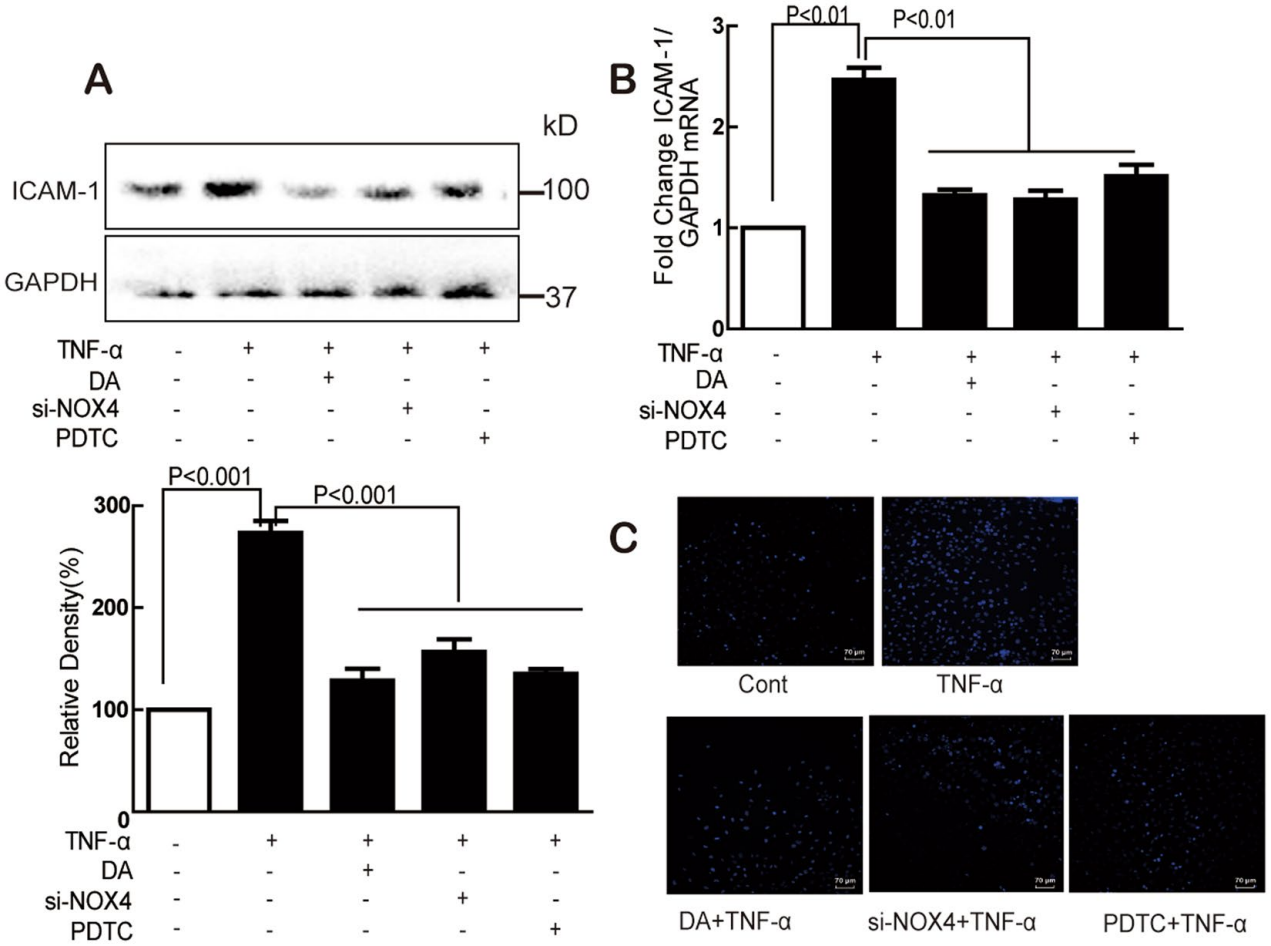
DA inhibited monocyte adhesion by decreasing ICAM-1 expression through NOX4/NF-κB signaling. Cells were treated with TNF-α (10 ng/mL) for 24 h with or without DA (10 nM), siNOX4, or PDTC (10 μM) pretreatment for 1 h, and the ICAM-1 protein (**A**) and mRNA (**B**) expression were determined by Western blot and RT-PCR; The adhesion of monocytes to endothelial cells was performed (**C**). Data were presented as mean ± SD of three independent experiments.

To explore the role of NOX4 and NF-κB in TNF-α-induced ICAM-1 expression, the effects of DA, NOX4 siRNA, or PDTC (a NF-κB inhibitor) were studied. The TNF-α-induced ICAM-1 expression was inhibited by DA, NOX4 siRNA, or PDTC at both protein (Fig. 8A) and mRNA (Fig. 8B) levels. Furthermore, all they can significantly reverse TNF-α-induced adhesion of THP-1 cells into HUVECs (Fig. 8C).

## Discussion

In this study, we investigated the inhibitory effect of DA on TNF-α-induced ICAM-1 expression in endothelial cells. The results demonstrated the following: (1) DA inhibited TNF-α-induced ICAM-1 expression; (2) ROS-dependent NF-κB signaling played an important role in this process; and (3) NOX4 served a dual role in regulating the TNF-α-induced ICAM-1 expression.

Consistent with previous reports^34,35^, our results showed that TNF-α was a potent ICAM-1 inducer in endothelial cells at both protein and transcriptional levels. Immunofluorescence results suggested that TNF-α induced the membrane expression of ICAM-1. The inhibitory effect of anti-ICAM-1 on the TNF-α-induced adhesion of monocytes to endothelial cells showed that ICAM-1 plays an important role in cell adhesion.

During ROS generation, a series of very small and highly reactive molecules, including hydroxyl radical (·OH), O_2_^•−^, H_2_O_2_, and peroxynitrite, act as signaling molecules to mediate various biological responses^36^. TNF-α-induced endothelial ROS production has been well established, but the detailed mechanisms remain unclear. In the present study, we found that TNF-α-induced ROS included both H_2_O_2_ and O_2_^•−^, which can be inhibited by both NAC and DA. Furthermore, DA showed no effect on DPPH free radicals. GSH comprises important intracellular antioxidants and antioxidative enzymes that help maintain the cellular redox homeostasis and protect cells from oxidative stress by directly scavenging ROS^37^. Interestingly, DA partly restored the TNF-α-induced decrease of GSH/GSSG ratio. Collectively, these data suggest that DA inhibited endothelial ROS formation. Accumulated data also suggest several sources of ROS involved in TNF-α-treated endothelial cells: NADPH oxidase, mitochondria, XO, and eNOS^38^. Under our experimental conditions, increased ROS was observed after TNF-α treatment, which was significantly suppressed by ROS scavenger NAC, NADPH oxidase inhibitor DPI, and DA. However, the inhibitors for the mitochondrial respiratory chain complex I-III, XO, and LOX showed no effects, suggesting that NADPH oxidase played an important role in this process. Considering that endothelial cells expressed several NOXs members^17,30,39^, the expression of NOX1, NOX2, and NOX4 was determined. The TNF-α-induced expression of both NOX2 and NOX4 contributed to the increased production of H_2_O_2_ and O_2_^•−^. However, DA significantly inhibited the expression of NOX4 without affecting NOX2. Thus, the inhibitory effect of DA on H_2_O_2_ and O_2_^•−^ might be merely ascribed to its effect on NOX4. The small transmembrane protein p22phox serves a scaffold function for the maturation and folding of the catalytically active NOX protein through a proline-rich region^40^. TNF-α induced the membrane expression of p22phox, which was suppressed by DA. Collectively, these results suggest that TNF-α induced NOX4 activation in HUVECs.

The role of NOX4 was further investigated. Jung *et al*.^41^ reported that blockade of TNF-α-induced ROS by NOX4 siRNA suppressed the TNF-α-induced ICAM-1 expression and subsequent monocyte adhesion to human aortic endothelial cells. Also our previous study showed that NOX4 plays an important role in the process of atherosclerosis^42^. In the current research, we found that the knockdown of NOX4 by siRNA significantly inhibited the TNF-α-induced ROS production and ICAM-1 expression at both protein and mRNA levels, as well as the THP-1**–**HUVEC adhesions. Interestingly, the inducible expression of ICAM-1 in response to TNF-α was abolished in NOX4-overexpressed endothelial cells, although the generation of ROS was enhanced. Furthermore, TNF-α showed no obvious effect on Nrf-2 nuclear translocation in normal HUVECs but increased its nuclear translocation in NOX4-overexpressed HUVECs. This finding suggests that TNF-α activated the Nrf-2 pathways when NOX4 was overexpressed. Along with the role of NF-κB (discussed below), these results suggest that NOX4 played dual roles in the TNF-α-induced ICAM-1 expression mediated by NF-κB and Nrf-2.

Transcription factors such as Nrf-1, Nrf-2, AP-1, and NF-κB have been reported to play important roles in TNF-α-induced ICAM-1 expression^43,44^. Most of these transcription factors are redox sensitive and can be activated by ROS^7,45^. Hence, the protein expression of NF-κB p65, Nrf-1, and Nrf-2 in the nucleus after TNF-α treatment was examined. Only the nuclear expression and nuclear translocation of NF-κB p65 were enhanced by TNF-α, and this upregulation was reversed by DA. IKKβ, whose activation results in IκBα phosphorylation, plays a critical role in the activation of NF-κB in the canonical pathway. The TNF-α-induced phosphorylation of both IKKβ and IκBα and the co-immunoprecipitation results suggest that their interactions were also enhanced. DA pretreatment partly reversed these alterations. Thus, these results suggest that TNF-α activated the NF-κB pathway by regulating IKKβ and IκBα. The inhibitory effect of DA suggests potential targets for this compound in the NF-κB pathway. The inhibitory effect of PDTC, an inhibitor of NF-κB, on the TNF-α-induced ICAM-1 expression and adhesion of THP-1 and HUVECs further confirmed the important role of NF-κB in the TNF-α-induced ICAM-1 expression.

**Figure 9:**
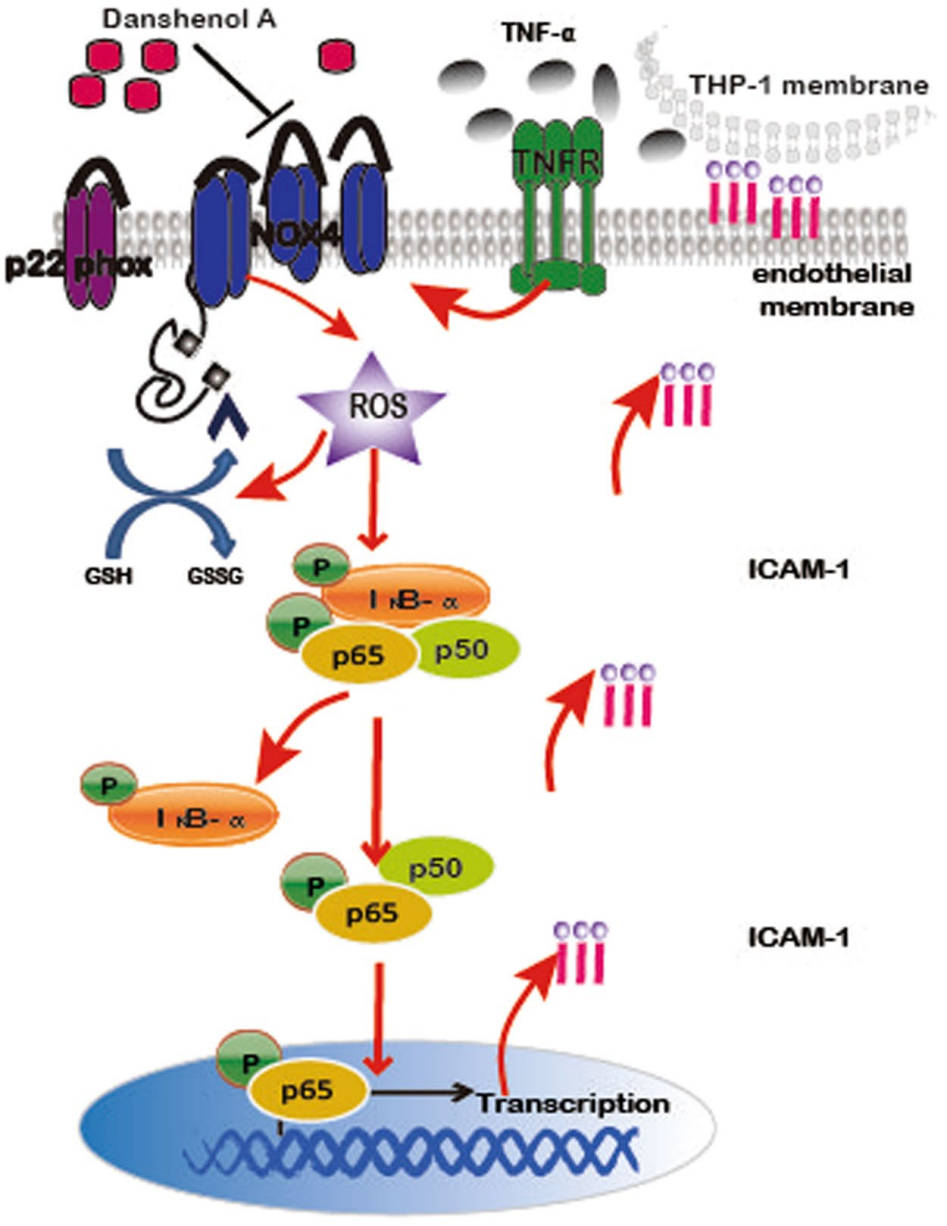
Schematic diagram illustrating the molecular mechanisms underlying the anti-ICAM-1 of DA in endothelial cells.

In conclusion, as depicted in Fig. 9, our results showed that DA, a natural product, inhibited TNF-α-induced ICAM-1 expression in endothelial cells. This inhibition was mediated by regulating the NOX4-dependent ROS generation and NF-κB activation. Furthermore, the results suggest that NOX4 played dual roles in regulating TNF-α-induced endothelial ICAM-1 expression, and the different expression statuses or patterns demonstrated distinct responses to TNF-α insult.

## Materials and Methods

### Chemicals, reagents, and antibodies

DA (>98%) was purchased from ChemFaces (China). Dimethyl sulfoxide (DMSO), 3-(4,5-dimethylthiazol-2-yl)-2,5-diphenyltetrazolium bromide (MTT), Hoechst 33342, 5-(6)-carboxy-2′,7′-dichlorodihydrofluorescein diacetate (DCFH_2_-DA), N-acetyl cysteine (NAC), diphenyleneiodonium chloride (DPI), rotenone (Rot), 2-thenoyltrifluoroacetone (TTFA), antimycin A (AA), allopurinol (All), nordihydroguaiaretic acid (NDGA) were purchased from Sigma-Aldrich (USA). Lipofectamine TM 3000, dihydroethidium (DHE), and Amplex Red were purchased from Life Technology (USA). Antibodies for NOX1, NOX2, NOX4, p22-phox, Nrf-1, and Nrf-2, Na^+^/K^+^ ATPase as well as protein A/G PLUS-Agarose were purchased from Santa Cruz (USA). The antibody for ICAM-1 was purchased from Abcam Company (UK). Antibodies for *p*-NF-κB p65, NF-κB p65, *p*-IKKβ, IKKβ, *p*-IκBα, IκBα, GAPDH, and Histone H3 were purchased from Cell Signaling Technology (USA). BCA protein kits were purchased from Thermo Fisher (USA). 2,2-Diphenyl-1-picrylhydrazyl (DPPH) was purchased from Aladdin (USA). siRNA for NOX4 was purchased from GenePharma Company (China). NOX4 overexpression plasmid was purchased from GeneChem Company (China). Primers and other materials for real-time PCR were purchased from Sangon Biotech (China) and TaKaRa Bio Group (Japan). P5 Primary Cell 4D-Nucleofector® X Kit L (24 RCT) was purchased from Lonza Company (Switzerland). GSH and GSSG assay kits were purchased from Beyotime Institute of Biotechnology (China). GKT137831 was purchased from BioChemPartner (China). FractionPREP^TM^ Cell Fractionation Kit was purchased from BioVision (USA). All other chemicals were purchased from Sigma-Aldrich (USA).

### Cell culture

HUVECs were cultured in F-12K medium with 1.5 g/L sodium bicarbonate, 100 µg/mL heparin, 2 mM L-glutamine, 30 µg/mL endothelial cell growth supplement and 10% FBS at 37 °C in a humidified atmosphere of 5% CO_2_. Tissue culture flasks, 96-well plates, and six-well plates were pre-coated with 0.2% gelatin. All assays were conducted using cells at 2–5 passages.

Human monocyte cell line (THP-1) obtained from ATCC (No. TIB-202, Rockville, MA) was cultured in RPMI 1640 medium containing 10% fetal calf serum, 2 mM glutamine, 100 U/mL penicillin, and 100 μg/mL streptomycin. Cells were seeded at 37 °C in a humidified atmosphere of 5% CO_2_ and 95% air.

### MTT assay

HUVECs were planted into 96-well plates (1 × 10^5^ cells/well) and treated with DA in series of concentrations. Cell viability was measured after 24 h of incubation followed by addition of 20 µL of MTT (5 mg/mL). After 4 h, the MTT-containing medium was slightly removed, and DMSO (100 μL) was added to solubilize the formazan. Absorbance at 570 nm was recorded using a Spectra Max M5 Microplate Reader.

### Monocyte–endothelial cell adhesion assay

HUVECs were pretreated with DA (10 nM), anti-ICAM-1 Ab (10 μg/mL), or other inhibitors for 1 h followed by TNF-α (10 ng/mL) treatment for 24 h. THP-1 cells labeled with Hoechst 33342 were co-incubated with HUVECs for 3 h in the dark at 37 °C. Non-adherent THP-1 cells were washed with PBS. Images of the adherent cells were obtained under random fields of each well by using IN Cell Analyzer 2000.

### Real-time PCR

Total RNA was extracted using TRIzol reagent. Quantitative real-time PCR (RT-PCR) was performed using SYBR Green PCR reagents (Applied Biosystems) with forward and reverse primers for ICAM-1 and GAPDH. The specific primers for ICAM-1 were 5′-GGCTGGAGCTGTTTGAGAAC-3′ (forward) and 5′-ACTGTGGG GTTCAACCTCT G-3′ (reverse). For GAPDH, the primers were 5′-CGAGATCCC TCCAAAATCAA-3′ (forward) and 5′-TTCACACCCATGGACGAACAT-3′ (reverse). Briefly, 1 μL of cDNA from the RT reaction was added to 10 μL of the RT-PCR mixture containing 5 μL of Master Mix and 0.2 μM forward and reverse primers. The samples were incubated at 95 °C for 2 min, followed by 40 cycles of reaction at 95 °C for 15 s and combined annealing step at 60 °C for 1 min. The samples were assessed by 2^−ΔΔCt^ relative quantitative analysis to determine the expression differences.

### Immunofluorescence assay

HUVECs (5 × 10^4^ cells/well) were seeded on glass slides in 12-well plates. After TNF-α treatment (with and without DA pretreatment), the slides were fixed with 4% PFA for 30 min. The slides were then permeabilized with PBST (containing 0.1% Triton x-100 in PBS solution) and blocked with PBS (containing 4% BSA in PBS solution). After incubation with primary antibody (1:1000) and secondary antibody (1:2000), cells were stained with Hoechst 33342 and DiO-C5-3 in the dark for 30 min. Fluorescence was observed using IN Cell Analyzer 2000.

### Detection of ROS, superoxide anion (O_2_^•**−**^), and H_2_O_2_

The production of O_2_^•−^, H_2_O_2_, and ROS was measured by DHE, Amplex Red, and DCFH_2_-DA, respectively. Briefly, confluent cells in six-well plates were pretreated with DA (10 nM), NADPH oxidase inhibitor DPI (1 µM), mitochondrial complex I inhibitor Rot (20 µM), mitochondrial complex II inhibitor TTFA (10 µM), mitochondrial complex III inhibitor AA (5 µM), xanthine oxidase inhibitor All (10 µM), or lipoxygenase inhibitor NDGA (10 µM). After TNF-α treatment for 30 min, cells were washed with PBS and incubated with DHE (10 μM), Amplex Red (50 μM), or DCFH_2_-DA (10 μM) in the dark at 37 °C for 30 min. Fluorescence was measured using Spectra Max M5 Microplate Reader or via flow cytometry by using a FACSCanto^TM^ system (BD Biosciences).

### Determination of GSH

HUVECs (5 × 10^6^ cells/well) in six-well plates were incubated with TNF-α for 1 h after DA pretreatment. GSH and GSSG contents were determined using commercial kits in accordance with the manufacturers’ protocols.

### DPPH assay

To evaluate the direct free radical scavenging effect of DA in a cell-free system, DPPH assay was performed as previously described^46^. Briefly, a series of DA concentrations (0.01 μM to 1 μM) was incubated with methanol solution of DPPH (5.0 × 10^−4^ M) and kept in the dark for 20 min. The absorbance of the samples was measured using a Spectra Max M5 Microplate Reader at 517 nm.

### Western blot analysis

The total proteins of the treated cells were collected. After quantitative determination of protein content, 30 μg of proteins was subjected to SDS-PAGE and then transferred onto PVDF membranes. After blocking with 5% nonfat milk in TBST (20 mM Tris-HCl, 500 mM NaCl, and 0.1% Tween-20) at room temperature for 1 h, the membranes were incubated with specific primary antibodies and secondary antibodies. Protein–antibody complexes were detected using an ECL Advanced Western Blot detection kit. The intensity of the bands was quantitated using Quantity One software (Bio-Rad).

### NOX4 knockdown

Approximately 1.0 × 10^6^/well cells were seeded in six-well plates overnight. Diluted siRNA and Lipofectamine^TM^ 3000 were mixed for 30 min at room temperature. Afterward, 200 μL of siRNA-lipofectamine complexes and 800 μL of Opti-MEM reduced serum medium were added to corresponding wells. The complexes were removed after incubation for 5 h, and cells were cultured with completed medium. The siRNA sequence for NOX4 was sense 5′-ACUAUGAUAUCUUCUGGUA-3′.

### NOX4 overexpression

HUVECs were transfected by electroporation with plasmid encoding GFP-NOX4. For each transfection, cDNA (3 μg) was dissolved in 82 μL of nucleofector solution and then added with 18 μL of supplemented P5 primary cell solution. Approximately 1 × 10^7^ to 1 × 10^8^ cells were resuspended in 100 μL of cDNA plus P5 primary cell solution and immediately electroporated in a 4D-Nucleofector X Kit L cuvette. Electroporated cells were transferred to six-well plates containing 2.5 mL of warm complete medium. Plates were cultured at 37 °C in a humidified atmosphere of 5% CO_2_. After 24 h, the medium was replaced with fresh medium at daily intervals.

### Immunoprecipitation

After determination of the protein concentrations, the cell extract was incubated with anti-IKKβ antibody (2 μg) for 2 h at 4 °C and then incubated with 20 μL of protein A/G plus-agarose beads overnight with constant shaking. Afterward, the beads were washed thrice with ice-cold radio immunoprecipitation assay (RIPA) buffer. The bound protein was extracted by adding 40 μL of 2 × SDS sample buffer and boiling for 5 min. The complexes were subjected to SDS-PAGE followed by Western blot.

### Statistical analysis

Data were expressed as means ± SD. Differences between groups were analyzed using Prism 5.0 (Graph Pad Software Inc, San Diego, CA), and statistical analysis was performed using one-way ANOVA, followed by Student–Newman–Keuls test. A value of *p* < 0.05 was considered statistically significant.
